# Roy Porter Student Prize Essay Figuring Pictures and Picturing Figures: Images of the Pregnant Body and the Unborn Child in England, 1540–*c*.1680

**DOI:** 10.1093/shm/hkx082

**Published:** 2017-10-24

**Authors:** Rebecca Whiteley

**Affiliations:** University College London, Department of History of Art, Gower Street, London, UK

**Keywords:** midwifery, childbirth, pregnancy, visual culture, print culture

## Abstract

Birth figures, or print images of the fetus in the uterus, were immensely popular in midwifery and surgical books in Europe in the sixteenth and seventeenth centuries. But despite their central role in the visual culture of pregnancy and childbirth during this period, very little critical attention has been paid to them. This article seeks to address this dearth by examining birth figures in their cultural context and exploring the various ways in which they may have been used and interpreted by early modern viewers. I argue that, through this process of exploring and contextualising early modern birth figures, we can gain a richer and more nuanced understanding of the early modern body, how it was visualised, understood and treated.

An earnest, cherubic toddler floats in what looks like an inverted glass flask. Accompanied by numerous fellows, each figure demonstrates a different acrobatic posture (Figure 1). These images seem strange to a modern eye: while we might suppose they represent a fetus *in utero* (which indeed they do), we are troubled by their non-naturalistic style, and perhaps by a feeling that they are rich in a symbolism with which we are not familiar. Their first viewers, in England in the 1540s, might also have found them strange, but in a different way, as these images offered to *them* an entirely new picture of a bodily interior that was largely understood to be both visually inaccessible and inherently mysterious. These images were published in the first print book in English to discuss pregnancy and childbirth, and to address itself to a female readership.

**Fig. 1:**
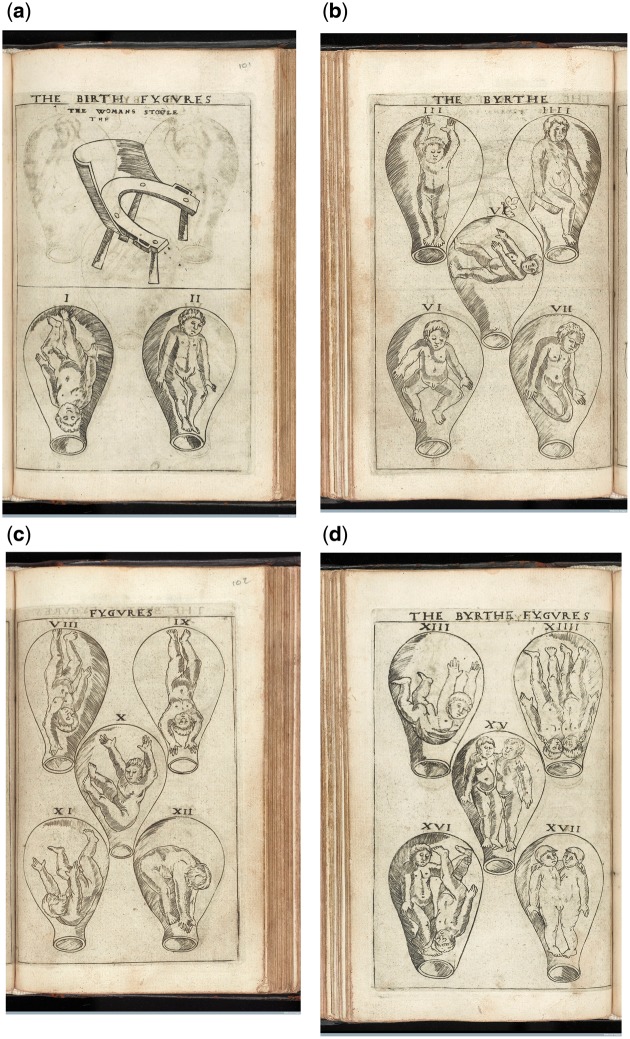
Anon. 1545. ‘The Byrthe Fygures’. Four Plates. Engraving. Plates: 10.5 × 15.7 cm. Printed in Eucharius Rösslin, The Byrth of Mankynde, Otherwyse Named the Womans Booke, T. Raynalde (trans), (London: Thomas Raynalde, 1545). Courtesy of the Wellcome Library, London. Copyrighted work available under Creative Commons Attribution only licence CC BY 4.0.

Originally published in German in 1513 by the physician Eucharius Rösslin, the book was translated into English under the title *The Byrth of Mankynde* by Richard Jonas (1540) and then again by Thomas Raynalde (1545). Although much of the medical content was not new in scholarly terms, this book and those that followed it radically changed the medical, cultural and social fabric of childbirth by introducing the subject to a broad reading audience for the first time: men and women, professional and lay. Over the next 250 years, childbirth went from an intensely private, all-female and essentially non-medical affair to a medical discipline presided over and publicly discussed by trained and professional male practitioners. These images were in print in midwifery books throughout this period of change, and as such they enable us to build a picture of how early modern people of all kinds, from pregnant women and midwives to surgeons and man-midwives understood, envisioned and treated the pregnant body and the unborn child.

These are images that depict the fetus in the uterus, but more than that, they are images that depict presentation, or the variety of postures a fetus can assume during labour. They range from the usual ‘cephalic’ or head-first presentation, to a variety of other difficult or undeliverable positions. Always printed in series, these images amount to an early modern system for understanding and categorising the possibilities of fetal malpresentation. Despite being widely reproduced in the later sixteenth and seventeenth centuries, these images have no established name or descriptor within scholarship. Lianne McTavish, for instance, calls them ‘images of the unborn’, Lyle Massey ‘the free-floating uterus’, Harold Speert ‘figures of the fetus in utero’ and K. B. Roberts and J. D. W. Tomlinson ‘figure[s] of the gravid uterus’.^1^ I propose, instead, that the term ‘birth figure’—used in *The Byrth of Mankynde*—might help scholars to think about these images as a unique iconographic group: distinct, in both composition and system of representation, from other images of pregnancy such as full-figure anatomical *gravida* figures.^2^

Rösslin’s book was enormously popular, among midwives and physicians, and also among the lay public. It was translated and published all over Europe and spawned the production of a whole genre of popular medical literature. In 1554, a Swiss surgeon Jakob Rüff published his own midwifery manual in Latin and German, *De Conceptu et Generatione Hominis* (1554). His book had newly commissioned birth figures and, like Rösslin’s work, was translated and published all over Europe (Figure 2).^3^ It is on the images found originally in these two works that this article will focus.^4^

**Fig. 2:**
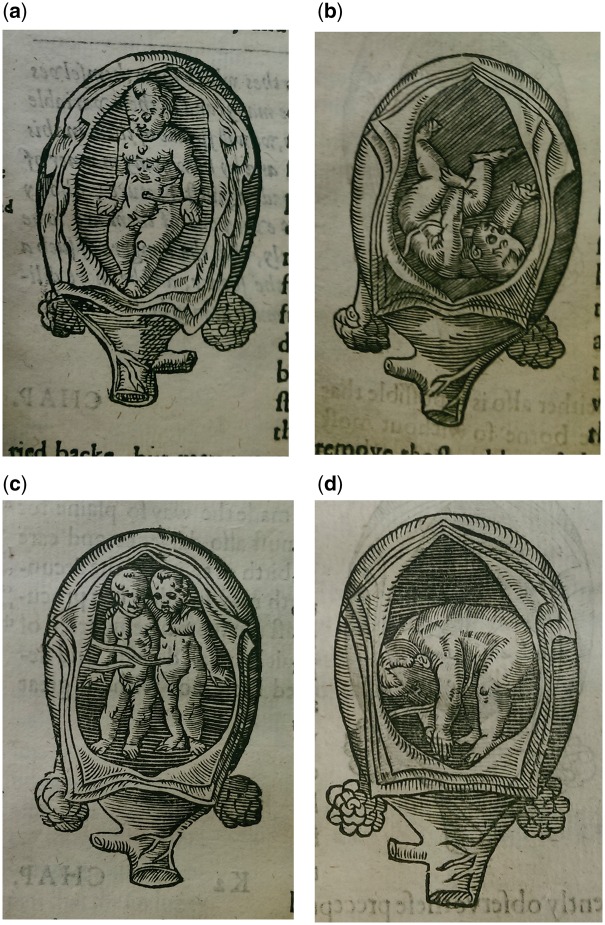
Anon. 1637. [four birth figures]. Woodcut. Figures: 4.5 × 7 cm. Printed in Jakob Rüff, The Expert Midwife or An Excellent and Most Necessary Treatise on the Generation and Birth of Man (London: E.G., 1637). Courtesy of the Wellcome Library, London. Copyrighted work available under Creative Commons Attribution only licence CC BY 4.0.

In England, Rösslin's book and his birth figures dominated the sixteenth century, Raynalde's translation going through 12 editions between 1545 and 1654. Rüff’s book, on the other hand, was only translated into English in 1637, and saw only one edition. However, his birth figures were appropriated by many other authors and came to dominate the visual culture of midwifery illustration after 1600 in England. Copies of Rüff’s birth figures were printed in at least six other midwifery titles, most of which went through multiple editions.^5^ While not every midwifery manual contained illustrations, many did and, until the late seventeenth century, most conformed more or less closely to the models of Rösslin and Rüff.^6^ Only with new developments in midwifery training and practice, and with the translation of new French manuals in the last decades of seventeenth century, did these two sets of birth figures begin to be overshadowed by new innovations in the genre. This article focuses on the period between 1540 and *c*.1680 in England because it was during this period that Rösslin and Rüff’s birth figures, and copies made of them, dominated the visual culture of midwifery manuals and, consequently, exercised a great influence over visual thinking about the pregnant body and the fetus *in utero*. As such, they both reflected and affected the period’s body culture and the ways in which people understood, visualised and treated the pregnant body and the unborn child.

While birth figures were widely disseminated as illustrations in midwifery and surgical books for several centuries, they have received almost no sustained attention from either historians or art historians. Historians of medical imagery have tended to employ a trivialising language, describing birth figures as ‘drawings of adult manikins, masquerading as fetuses ‘bottled-up’ inside the uterus, as ‘bizarre portrayals of fetuses *in utero*’ and as ‘wildly fantastic pictures’.^7^ Such texts tend to situate birth figures in a narrative of increasingly ‘accurate’ and ‘naturalistic’ portrayals of the bodily interior, comparing them, to their detriment, both with Leonardo da Vinci’s anatomical drawings, and the eighteenth-century anatomical illustrations commissioned by William Hunter and William Smellie.^8^

Some historians of midwifery and the body have, however, in recent decades, approached birth figures in less teleological and more historically sensitive ways. Some have, for instance, incorporated birth figures into feminist analyses of how power, agency and knowledge was lost by midwives to doctors and male practitioners during the early modern period. Such histories have tended to emphasise birth figures as images that functioned for male practitioners to deny the agency and the importance of the female body in birth, and to establish the *fetus* as the primary patient.^9^ I term this interpretation the ‘maternal erasure’ theory, as scholars such as Karen Newman and Eve Keller interpret the disembodied uterus as a statement of the irrelevance of the rest of maternal body to the practice of midwifery, and the importance of the fetus as individual. I will argue later in this article, however, within an early modern context, that the ‘maternal erasure’ interpretation can give at best a partial, and at worst an anachronistic, view of how such images were used and understood.

Other historians have suggested that birth figures are either ‘mnemonic’ devices, according to Mary Fissell, or ‘diagrams’, according to Elaine Hobby, that help midwives to understand and remember the variety of fetal malpresentations.^10^ While such interpretations may be more historically sensitive, neither historian interrogates or substantiates their analysis. Indeed, to my knowledge, the only scholar to treat these images at length is the art historian Lianne McTavish, who argues that ‘regarding images of the unborn as diagrams fosters a more historical understanding of them, while providing insight into how they actively produced meaning’.^11^ McTavish argues that birth figures were never intended to *look like* the interior of the body, but were instead images that helped practitioners to understand the invisible interior and practice upon it.

In this article, I will expand on McTavish’s argument, proposing a more detailed model for *how* the birth figure as diagram or key might have worked for early modern midwives. But I will also propose that neither ‘maternal erasure’ nor ‘diagrammatic’ analyses tell the whole story of these images, that in fact birth figures also engaged with parts of early modern body culture that are largely overlooked today. In my approach to these images I have followed Michael Baxandall’s work on the ‘period eye’, which attempts to look at images as they would have been seen by a contemporary viewer, through the lens of the culture in which they were produced. Such an approach, as Baxandall argues, assumes that ‘social history and art history are continuous, each offering necessary insights into the other’.^12^ I, therefore, approach birth figures as historical primary material: establishing a broad social and cultural context for these images and building a ‘period eye’ with which to look at them, and thus interrogating what they themselves can tell us about the culture that produced them. Baxandall argues of the historic image that ‘one has to learn to read it, just as one has to learn to read a text from a different culture, even when one knows, in a limited sense, the language’.^13^ This article will propose a ‘cognitive style’ for reading birth figures that does not rely exclusively on the context of new anatomical discoveries and the progression of obstetrical technique. Rather, to ‘read’ birth figures in their own ‘language’, I will look at analogical, alchemical and humoral systems for thinking about the body, as well as considering the way in which they were employed not only by practitioners and learned physicians but also by midwives and the women they cared for.

Baxandall’s argument, that the image must be understood within the framework of its own culture, chimes with another important methodological proposition for this article: Barbara Duden’s work on ‘body history’. While Baxandall argues that images are a product of their culture and must not be read anachronistically through the framework of our own, Duden makes a similar argument with regards to the body itself. She points out that too often the body is understood as a biological truth and a historical constant—the same in 1600 as it is today. Duden’s work challenges the idea of the body as a ‘natural given’ and instead attempts to look at it as a product of a period’s culture.^14^ Following her example, and using birth figures as primary historical material, I will here attempt to establish ‘the reality-generating experience of the body that is unique and specific to a given historical period’.^15^ I will demonstrate that the early modern body was the locus of multifarious and imaginative thinking not yet pinioned by ideas of physiological ‘truth’.

Birth figures represented the body and functioned for viewers in various ways: from providing diagrammatic keys to midwives, to speaking about the rich culture of early modern analogy, to working wonders upon the mother’s body. In this article, I will approach birth figures as primary historical evidence for a complex body culture, and make an argument for each of these functions. I will argue, finally, that their long lives and popularity in print indicate their perceived usefulness among early modern readers, and that, as such, they can tell us about elements of early modern body culture that are too often overlooked. In this approach, I have taken inspiration from Mary Fissell’s article on the frontispiece image common to many editions of *Aristotle’s Masterpiece* (1684). Fissell argues that ‘The image had many referents, and we need to consider multiple meanings and open-ended interpretations if we are to understand how the picture functioned.’^16^ It is this attention to multiplicities of function that this article brings to the discussion of birth figures.

## Anatomy and Practice

**Fig. 3:**
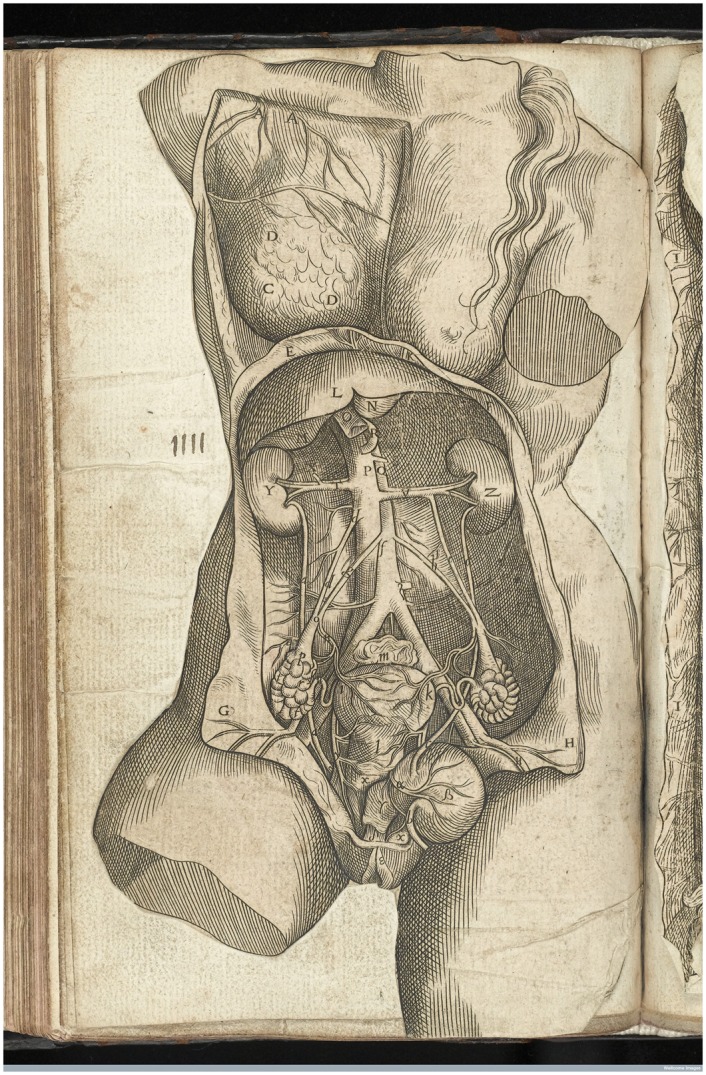
Anon. 1543. [Anatomical image of the female urogenital system]. Engraving. Figure: 10.5 × 18.5 cm. Copied from Andreas Vesalius, De humani corporis fabrica libri septem (Basileae: I. Oporini, 1543), printed in Eucharius Rösslin, The Byrth of Mankynde, Otherwyse Named the Womans Booke, T. Raynalde (trans), (London: Thomas Raynalde, 1545). Courtesy of the Wellcome Library, London. Copyrighted work available under Creative Commons Attribution only licence CC BY 4.0.

Sachiko Kusukawa, in *Picturing the Book of Nature*, warns that ‘Pictures are of course visual, but understanding what exactly they have to do with observation or description requires careful investigation.’^17^ In the case of birth figures, it is tempting to assume that they formed part of the rising ‘epistemic genre’ of *observatio*, which, as Gianna Pomata describes, produced ‘a new self-consciousness on the part of the anatomical observer’ in the sixteenth century.^18^ But birth figures actually date back to at least the ninth century, to gynaecological manuscripts by the fifth- or sixth-century author Muscio.^19^ In all of this long history, the images were never presented as anatomical, as primarily informed by dissection or as describing the positions and qualities of the human organs. Instead, in depicting fetal presentation, these images are imagined, highly selective views of a *living* bodily interior that is in the process of labour. As early as 1545, with the new translation of *The Byrth of Mankynde* by Thomas Raynalde, midwifery manuals embraced the contemporary in anatomical research. This edition included images copied from Vesalius’ *De Humani Corporis Fabrica* (Figure 3).^20^ First published only two years before, by 1545 Vesalius’ anatomical images were rapidly being disseminated all over Europe. Included in the midwifery manual alongside birth figures, the two kinds of illustrations demanded different systems for interpretation. As Kusukawa has argued, Vesalius’ anatomical images were produced to aid students in reading dissections—they are keys to and descriptions of the opened body, teaching the initiate how to make sense of the actual body, to locate it within medical and physiological theory.^21^ The editor of *The Byrth of Mankynde* felt similarly about these images, describing their effects on the viewer ‘as thoughe ye were present at the cuttynge open or anathomye of a ded woman’.^22^

Birth figures were not included under this claim. Indeed, for Raynalde, it was not remarkable that some images of the body should be anatomical and some not. Birth figures were working in a different register, imagining the bodily interior and depicting it living and in process. As Barbara Duden has pointed out, at this time, ‘the dead body did not yet cast its shadow on the living body’.^23^ As both she and Michel Foucault have argued, knowledge gained through dissection, giving a concrete physiological picture of the inside of the body, did not come to dominate how the living body was visualised until the nineteenth century.^24^ In the early modern period, anatomical images and birth figures could function side by side without contradiction; Vesalius’ anatomy could picture the dead body, and birth figures the living. Daston and Galison have argued that ‘Epistemic virtues do not replace one another like a succession of kings. Rather, they accumulate into a repertoire of possible forms of knowing.’^25^ The anatomical image and the birth figure provided different ways of knowing the bodily interior, and they formed part of a growing understanding of the body in which one kind of knowing added to and influenced, but did not render obsolete, the other.

After the 1545 edition of *The Byrth of Mankynde*, anatomy was regularly included in midwifery manuals, where it was presented as a kind of theoretical grounding for midwives. In *The Byrth of Mankynde*, for instance, anatomy was described as ‘the foundation and ground, … the better to understand how every thyng cummeth to passe within your bodyes in tyme of conception, of baryng, and of byrth’.^26^ But this knowledge was understood as peripheral to the main art and skill of midwifery, and as most midwives had no access to education in anatomy, it was, in practical terms, not a necessity. Indeed, even as late as 1737, the midwife and author Sarah Stone was still describing her attendance at public anatomies, and her reading of anatomical books as useful, but not a central pillar of the discipline. She states, moreover, that without extensive practical experience, anatomical knowledge ‘would have signified but little’.^27^

In midwifery manuals, anatomical images and text were invariably restricted to a prefatory section. They gave a highly restricted taste of the kinds of academic knowledge that were the province of male physicians. Birth figures, on the other hand, were often printed interspersed throughout the main body of the text, referring to the living body in labour with which midwives dealt in their daily practice. While the fetuses in anatomical images often look sombre, still, secretive, even totemic, birth figures present a fetus which is active, open and, taken as a series, various, changeable and acrobatic. Birth figures engaged with the living variability of the body, while anatomical images rendered it static, regular and typical. Certainly, in a birth figure the uterus is opened and excised from the body, but it is an imagined excision and an imagined cut, as the fetus is still alive and lively, ‘swimming’ in the amniotic fluid that is retained, despite the opening through which we look. Anatomical detail is subordinate to the depiction of fetal presentation. While birth figures and anatomical images were regularly collected together in midwifery manuals, they were practically and perceptually apart. The anatomical was static, theoretical and academic, while birth figures were active, practical and ‘practitional’.^28^

Despite advances in anatomy, the labouring body was still profoundly mysterious and essentially unseeable in the early modern period. It was a realm completely outside the province of the midwife’s art. Before visualising technologies and widespread detailed physiological knowledge, midwifery rarely included attempts to understand or intervene in the *interior* processes of birth. Both midwifery manuals and later accounts of practice by English midwives, including those of Percival Willughby and Sarah Stone, suggest that most midwives restricted their practice to the bodily exterior and were often unable to visualise what has happening inside.^29^ One seventeenth-century diarist, Alice Thornton, for instance, recounted one of her own labours in which her ‘sweete goodly son was turned wrong by the fall I gott in September before, nor had the midwife skill to turne him right, which was the cause of the losse of his life, and the hazard of my owne.’ ^30^ She reports a case in which it was known that the fetus was positioned wrongly for birth, but that the midwife did not have the skills in visualising the position or turning the child to put it right.

As has been noted, this is not especially surprising, as most babies, presenting in a cephalic or ‘head-first’ presentation, will be born spontaneously and can simply be received by the midwife. In this period, therefore, the midwife’s main tasks included care and encouragement of the mother, the administering of various receipts and medicines, and after birth, the cutting of the umbilical cord, delivery of the placenta and washing and swaddling of the fetus.^31^ Even in cases of malpresentation, where the fetus presented another part of the body and often could not be born spontaneously, the usual remedy was to push back the presenting part of the fetus and to toss the mother around in the hopes of shaking the fetus into the ‘natural’ cephalic presentation.^32^ I argue that this approach to the labouring body and to midwifery practice meant that the midwife was almost never required to visualise the exact position of the fetus, or to intervene inside the body.

However, I also argue that, in the sixteenth century, the publication of midwifery manuals, and particularly of birth figures, began to offer a different way to visualise the body, and a different way to practise midwifery. Podalic version, which involves finding the feet of a malpresenting fetus and pulling it out by them, presented an entirely new conception of the body and of childbirth. The method was first published by the French surgeon Ambroise Paré in the mid-sixteenth century in France, and was slowly disseminated in text and practice throughout the seventeenth century in England.^33^ This intervention offered a new way to deliver obstructed fetuses who would otherwise have been delivered using craniotomy, which would certainly, or dismemberment, which would likely, kill them. But it also offered a completely different way of looking at childbirth. No longer was the aim to return the body to a state of unseen and independently functioning ‘naturalness’, but rather to actively intervene in its processes, and to deliver the baby in an ‘unnatural’ yet much more convenient presentation. However, to do this, the practitioner had to engage with a process of concretely visualising the body. First, they would have to establish the exact position of the fetus, what presented at the cervix and where the feet were, then they would have to move their hand *inside* the uterus to manipulate the fetus’s position and exert traction on its feet and legs, in order to effect delivery. Midwives were no longer attendants at a natural, internal and unknowable process, now they were agents in a new way, intervening in, correcting and ameliorating the body’s processes.

This ideal of ‘natural’ labour should be understood within the context of Hannah Newton’s argument that ‘Nature’, in this period, was an active and personified agent within the body who helped to rebalance the humours and purge disease.^34^ Within this framework, the midwife’s job would have been to aid that Nature in her processes, in this case, by returning the fetus to its ‘natural’ position. This commitment within physic to aiding Nature may explain why reduction to the head was so often advised, and why podalic version took so long to disseminate.

Another reason for the slow dissemination of podalic version must be that it required, first, an entirely new framework for perceiving the body. As Jakob Rüff wrote in his manual, the midwife’s duty was now to ‘gently apply her hands to the worke as she ought, by feeling and searching with her fingers how the child lieth’.^35^ By feeling what part of the fetus presented at the cervix, midwives were learning how to concretely establish the position of the fetus. And for these practitioners, I argue, birth figures formed a kind of visual training and a ‘practitional’ key. Midwives could map the scant tactile information they had gathered from a labouring woman’s body onto the series of images in a midwifery manual. Doing so, a midwife would develop and fill out her picture of the fetus and thus how she might rectify its presentation.

It is impossible to establish whether (and how many) midwives did read birth figures in this way, because so few recorded their experiences of reading, or of learning and practising their trade. However, Jennifer Richards has cited evidence for English early modern midwives as ‘thoughtful, practical readers’—whom she contrasts with both learned male readers and ‘ill-informed’ female midwives—in Edward Poeton’s manuscript *The Midwives Deputie* (*c*.1630s).^36^ Moreover, this process of learning though images is described in the manual of the German midwife Justine Siegemund. Siegemund was a remarkable midwife, first because she published a treatise in a field almost entirely dominated by male authors, and secondly because she learned her profession not through the traditional system of apprenticeship and practical experience, but from books. Her understanding of the body and of midwifery practice are, therefore, extremely important in understanding how books and their images were understood and used by early modern readers.^37^ Siegemund initially began to read about midwifery to satisfy her own desire for knowledge after suffering a traumatic misdiagnosis of pregnancy as a young woman. She later became a practising midwife when local women and midwives, aware of her reading, asked her to consult on difficult labours. In her book, Siegemund describes the first case she was called to attend, one of arm presentation:

The midwife, that is, her sister-in-law, entreated me, for the love of God, to advise them because she had seen me with books with illustrations of sundry births. So I got out the books and looked to see what postures were depicted there. Because, however, it was impossible for this midwife to determine which picture corresponded to the posture of the laboring woman’s child, they despaired.^38^

Siegemund, however, delivered the child and, winning the confidence of the local midwives, began to be regularly called to difficult births. As she gained practical experience, she also honed the skills that allowed her to reconcile actual labours with the presentations depicted in birth figures, becoming more and more able to visualise and rectify malpresentations. Siegemund’s narrative describes a community in the middle of great perceptual and ‘practitional’ changes. The midwives were aware of the usefulness of birth figures, and of books more generally to their practice. Indeed, all were agreed that birth figures might be useful if they could be matched with the malpresentation in question. Yet the midwife with her traditional ‘practitional’ understanding, and Siegemund with her knowledge exclusively from texts and images, could not readily reconcile the two. It was Siegemund’s continued practice, combining practical and textual knowledge, that allowed her more and more easily to enact this reconciliation, and so more easily deliver obstructed births. Eventually, she developed her own podalic version and, when committing her knowledge and experience to a treatise, produced her own birth figures. Siegemund’s text is largely written as a dialogue between herself and a young midwife-pupil, and, in a remarkable meta-narrative, those engravings that are published in the book are also provided, within the narrative, to the fictional pupil as learning aids. This pupil gives voice to Siegemund’s own deep conviction in the ability of the birth figure to teach practice: ‘I can grasp it better by looking at a copper engraving together with a detailed report than from the report alone. The copper engravings light up my eyes as it were and place understanding in my hands.’^39^

Presumably, many other midwives and surgeons who did not produce treatises went through a similar process of learning to reconcile the body, text and image in the pursuit of good practice. It is important to note, however, that in England especially, where there was no formal system for midwifery training, such perceptual and practical skills can only have developed very slowly and sporadically: only where midwives who were willing to change their habits met with books and those able to interpret them.

I propose that birth figures disseminated a new way of thinking about the bodily interior; they gave the women who saw them a visual system for thinking about the opaque, mysterious and troubling pregnant body. And within this context, many of their features come into focus. The uterus is balloon-like because it is simply meant to locate the fetus in relation to the cervical opening. The fetus itself is small and spread out to give clarity to an operation which, in reality, is ambiguous, difficult and cramped. The fetuses here seem to perform—limbs akimbo, they *show* the viewer how they are positioned. This is not merely a strange quirk of early modern bodily representation, but a system that allows midwives to more clearly understand, remember and apply various presentations to cases they encountered. Rather than being naive or outdated anatomies, therefore, birth figures should be understood as ‘practitional’ images which engaged with the living, individual body and facilitated the development of new ways of picturing and practising on that body.

## The Analogical Body

Apart from being ‘practitional’ images—ones that functioned practically for midwives and, as I have argued, helped to develop a new way of visualising the living bodily interior—birth figures interacted with established early modern body culture more widely. As well as projecting a more concretised view of the bodily interior, birth figures interacted iconographically with ancient and deeply pervasive analogical thinking about the body.

As Foucault has discussed in *The Order of Things*, resemblance was the fundamental system upon which things, and their relations, were understood in the early modern world. ‘The universe was folded in upon itself: the earth echoing the sky, faces seeing themselves reflected in the stars, and plants holding within their stems the secrets that were of use to man.’^40^ Analogy was a way of seeing different realms of the universe as fundamentally connected, reflective of each other. Through analogy, knowledge of one could give knowledge of the other.

One of the most pervasive early modern analogies was between the world at large, or the macrocosm, and the human body, or the microcosm. This system allowed people to comprehend those things that were not readily accessible—like the bodily interior. It also placed the human body at the centre of a universe of concentric rings of resemblance, each connected to the other and thus speaking of an overarching order. This analogical system was also often expressed in early modern imagery. As Foucault asserts, ‘It was resemblance that largely guided exegesis and the interpretation of texts; it was resemblance that organized the play of symbols, made possible knowledge of things visible and invisible, and controlled the art of representing them.’^41^

Birth figures were no exception to this fundamental tenet of early modern knowledge, and analogy is central to how they speak about the body. These are images that can only be understood in the context of an early modern body culture that placed at least as much emphasis on the analogical body as it did on the anatomical. Resemblances in birth figures range from the alchemical flask, to fruit trees and domestic spaces. Each analogy spoke about a different aspect of the pregnant body, connecting the invisible bodily interior with other kinds of more accessible knowledge.

It was common in the early modern period for the uterus to be associated with all kinds of pots and vessels. Thomas Laqueur has noted the handled vase in the foreground of one of Charles Estienne’s erotically charged female anatomies, arguing that ‘It too may represent the womb—the uterus with handles as “seminal vessels” and the bearded men as ovaries—both linguistically and because of its shape (Latin *vas*, French *vase*, container or vessel)’.^42^ Laurinda Dixon has made a similar argument about the small charcoal burners often depicted in seventeenth-century Dutch genre paintings of sick women. ‘Given common contemporary references to the womb as a vessel and the female abdominal cavity as a box (a slang term that is current today), this image becomes a conspicuous visual equivalent for the heated, displaced womb implicated in *furor uterinus*.’^43^ Dixon argues that pots were a common symbol for the uterus, and thus a broken pot a symbol of birth, miscarriage or loss of virginity. The open and yet enclosing nature of such vessels has obvious associations with the uterus, symbol *par excellence* of the ‘leaky’ female body. In such paintings as Dixon describes, these charcoal burners draw the viewer’s attention to the hidden subject of the painting: the uterus, and they are a kind of medical prognostic: the uterus has moved out of its normal place and is causing problems.

**Fig. 4:**
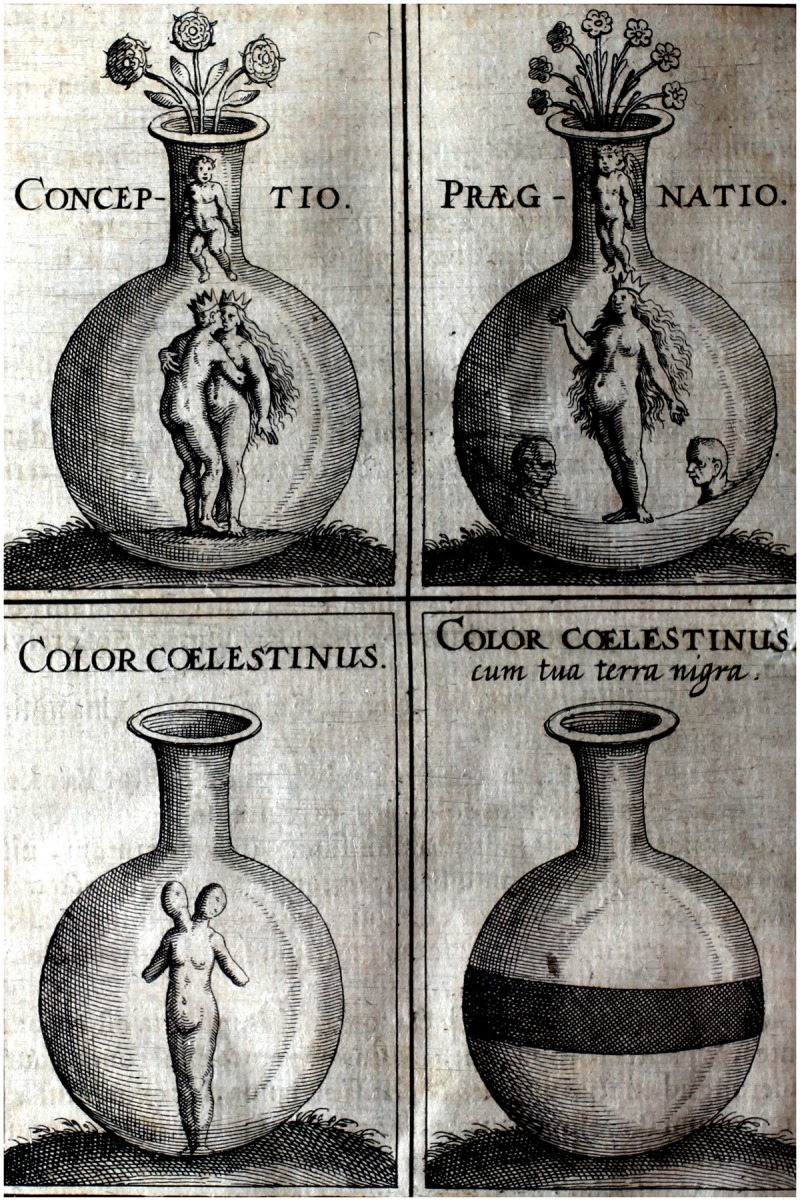
Anon. 1628. [Alchemical figures]. Engraving. Plate: 10.5 × 14.8 cm. Printed in Johann Daniel Mylius, Joannis Danielis Mylii philosophiae & medicinae doctoris Anatomia auri, sive Tyrocinium medico-chymicum, continens in se partes quinque… (Frankfurt: Lucae Iennisi, 1628). Photo: Warburg Institute (The Warburg Institute Iconographic Database). This material is licensed under http://creativecommons.org/licenses/by-nc/3.0/ \t blank Creative Commons Attribution-Non Commercial 3.0 Unported License.

Given the general understanding of the uterus as a vessel in this period, and the specific use of pots and vessels to represent the uterus in images of various kinds, it is likely that Rösslin’s birth figures would also have been understood as actively associating the uterus with the vessel (Figure 1). Indeed, the uterus here looks like nothing so much as a round-bottomed glass vessel or flask—a piece of equipment that would have been familiar to some early modern viewers from the iconography of alchemy. And just as the uterus in the birth figure resembles a flask, in alchemical imagery the flask is often associated with the uterus by the placing of an infant, often representing the white stone, inside it (Figure 4). The uterus and the alchemical flask were analogous because they were both the sites of generative processes. In the alchemical flask, sulphur and mercury would be mixed and heated in a symbolic marriage and consummation. The product, or conception, would be the white stone, the first step in making the philosopher’s stone. In alchemical imagery, these three substances were often anthropomorphically represented as a male sulphur, a female mercury and the white stone as their child. In the uterus, according to some ideas of conception, a similar process was enacted. The male and female seeds met, mixed and were heated and contained by the uterus until they formed something finer and purer than the sum of their parts—a child. The surgeon Ambroise Paré, for instance, describes conception in very alchemical terms: ‘the male and female yeeld forth their seeds, which presently mixed and conjoyned, are received and kept in the females wombe. For, the seed is a certaine spumous or foamie humour replenished with vitall spirit, by the benefit whereof, as it were by a certain ebullition or fermentation, it is puffed up and swolne bigger’.^44^

While it was common for alchemists to think analogically about their work in terms of human conception, some also felt that human conception itself was within their purview. William Newman, in his book *Promethean Ambitions*, describes how alchemy was felt to be an art that had dangerous powers to exceed or better the processes of nature.^45^ The earth naturally created precious metals, and the uterus the child, but alchemy presented the possibility of doing both of these things artificially, better and faster, in the workshop. Some alchemists, including Paracelsus, believed that it was possible to grow a living human fetus, or homunculus, from semen in the alchemical workshop, using a glass flask as a pseudo-uterus. This belief stemmed from a slightly different idea of conception, in which all the faculty for forming and creating resides in the semen, which then acts on the matter of blood or seed in the female uterus to make a child. Within this system, it was entirely feasible for the wonderfully creative semen to produce a child without the female uterus—such children might even be better, purer or more powerful, because they were not subject to the female body’s corrupting influences.^46^

In each of these systems—the symbolic generation of the child as white stone, and the actual but mysterious and dubious creation of the homunculus in the alchemist’s glass flask—the resultant images bear strong resemblances to Rösslin’s birth figures (Figure 1). Different learned readers might see different specifics in the image, but all interpretations are connected by the web of analogical thinking, which saw the same creative faculty in the uterus, in the world, and in the alchemist’s flask. The birth figure—whether it was understood as literal, symbolic or analogous, or, as I think most likely, all of these at once—was an expression of the creative faculty of the universe.

But for those viewers who were unfamiliar with the iconography of alchemy, there was another vessel analogy that would have been almost universally unmistakeable. If Rösslin’s birth figures looked like the round-bottomed glass flasks used by alchemists, they also looked like a very similar flask used by uroscopists. Uroscopy, or the examination of urine, was one of the most widely used diagnostic tools of the early modern era; the colour, consistency and sediments in urine were understood as indicators of sickness in the body, and one thing uroscopy was widely understood to predict was pregnancy. Michael Stolberg has recorded that uroscopy was particularly valued as a test for pregnancy that did not rely on the testimony of the mother, who might misinterpret or misrepresent the sensations she felt.^47^ In many Dutch genre-paintings of the period, the urine flask is the focus of a scene in which a weeping young woman, often accompanied by angry relatives, has her pregnancy confirmed by the uroscopist. In such paintings, the urine flask is a visible and unequivocal test for something that was otherwise inherently unknowable, mysterious and troubling. It not only stood in for the uterus, indicating its centrality to the image, it also *spoke* about the hidden organ, diagnosing and displaying its condition. This diagnostic capacity is visualised with remarkable literalism in the painting *The Doctor’s Examination* by Godfried Schalcken (*c*.1690). In this image, the weeping woman, the angry father and the obscene gesture made by the grinning boy all point to a pregnancy out of wedlock. And the viewer’s diagnosis is confirmed in the urine flask, where fine white sediment forms itself into the shape of a child. Such a sight would not have been strange or incongruous to the early modern viewer, for whom the uterus and flask were analogous, and for whom the urine flask specifically was understood to represent and diagnose the uterus. The central focus of the painting, that which the doctor holds up to the light for all to see, is both the diagnostic test and the condition it confirms—the flask and the uterus simultaneously.

Rösslin’s birth figures, too, seem to flicker between test and embodiment, between the pregnant uterus and the flask of urine. Stolberg has argued that uroscopy in the early modern period was a well-respected and reliable diagnostic practice, one which gave certainty to mysterious internal bodily conditions. The casebooks of physicians Simon Forman and Richard Napier confirm that women regularly sent their urine to be tested for pregnancy, hoping to get a definitive answer well before other physical symptoms could be trusted.^48^ As such, linking the urine flask with the birth figure would imbue the image with a kind of medical pedigree and certainty. It associated the new midwifery texts with well-known practices in physic, arguing for the image’s authority over the unseeable body.

If the uterus/vessel analogy spoke to the early modern viewer about conception and about the early identification of pregnancy, then another school of analogy spoke about fetal development and childbirth. For most early modern people, pregnancy was most closely analogous with the daily tasks of rural and agricultural life. Cyclical processes such as ploughing, sowing and harvesting, as well as the traditionally female duties of housekeeping and hospitality, were regularly used as frameworks for understanding pregnancy and birth.

**Fig. 5:**
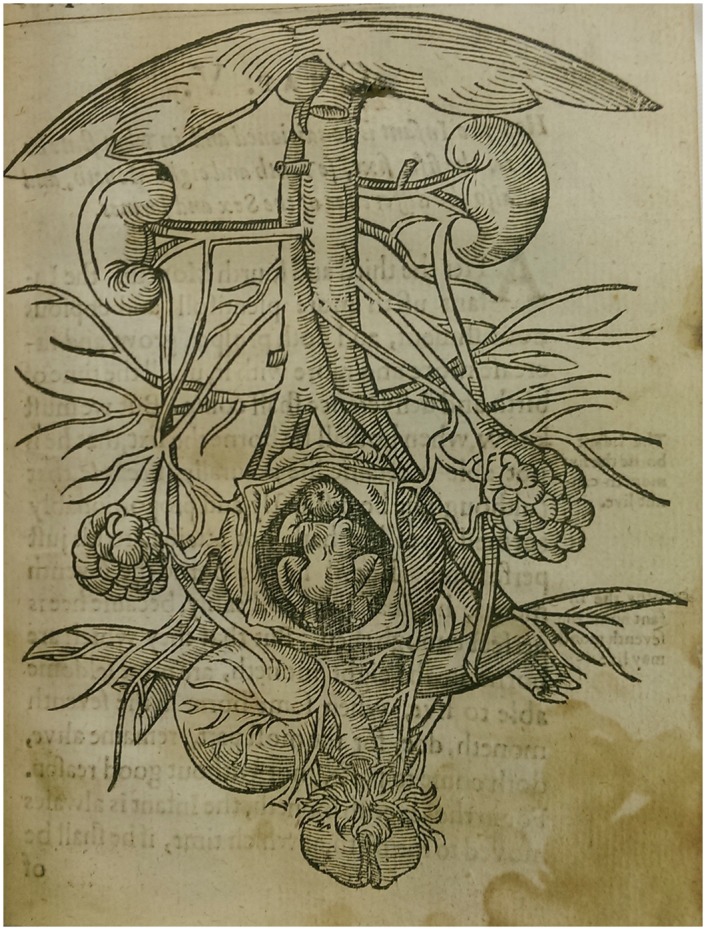
Anon. 1637. [Anatomical image of the female urogenital system]. Woodcut. Figure: 11.5 × 15 cm. Printed in Jakob Rüff, The Expert Midwife or An Excellent and Most Necessary Treatise on the Generation and Birth of Man (London: E.G., 1637). Courtesy of the Wellcome Library, London. Copyrighted work available under Creative Commons Attribution only licence CC BY 4.0.

This is more immediately obvious to the modern viewer in Rüff’s birth figures, in which the bodily interior is explicitly vegetal; the membranes of the uterus peeling away like the rind of a fruit to reveal the fetal flesh within (Figure 2). In his anatomical images too, the analogy with flora holds sway, with the veins becoming the trunk of a bodily tree, the organs hanging like fruits (Figure 5). Within this internal landscape, the fetus is simultaneously the ‘fruit’ (a common verbal as well as visual analogy for children at this time) and a miniature figure dwelling, hermit-like, in the maternal/arboreal environment. From farmers to physicians, this kind of verdant analogy was a powerful tool for thinking about the body.

Percival Willughby was an English surgeon and man-midwife operating in the middle decades of the seventeenth century. He mostly attended obstetrical emergencies, wherein he assisted regular midwives with a range of complications from fetal malpresentation to uterine haemorrhage. He was perhaps the first practising English midwife to write a midwifery manual. Although his manuscript was not published until it was rediscovered in the nineteenth century, it gives a valuable picture of English midwifery in the seventeenth century. Willughby repeatedly uses verdant analogy to explain the logic of his practice: ‘Let all midwives observe the wayes and proceedings of nature for the production of their fruits in trees, the ripening of walnuts, and almonds, from their first knotting, unto the opening of the husk, and falling of the nut, and considering their signatures, to take notice, how beneficiall their oiles may bee for use in their practice, for the easing of their labouring woman.’^49^

The midwife is enjoined to look carefully at the natural world, and specifically to look for signatures that related to the pregnant and labouring body. Here Willughby links the process of pregnancy and labour with the ripening of nuts. The doctrine of signatures taught that such analogies could be employed to produce medicines: the nut’s sympathies with the uterus made nut oils an excellent treatment in labour, presumably as a lubricant, for which most midwifery authors recommended various fats and oils.

Indeed, Willughby’s use of this analogy went both ways. If knowledge of the nut’s resemblance to the belly could produce medicine, then knowledge of the *belly’s* resemblance to the *nut* could also dictate good medical practice. Of the process of labour he writes: ‘as the fruit ripeneth, so, by degrees, this husk, of it self, will separate from the shell, which, at last, by it’s own accord, chappeth, and, with a fissure, openeth, and, by degrees, separateth from the fruit. Then doth the husk turn up the edges, and give way, without any enforcement, for the falling off the nut.’^50^ At the time of Willughby’s writing, there were competing theories about how to ensure the safest labour. One held that the quicker the labour was, the better, and some authors, including Rösslin, advised that the midwife manually dilate the vagina and cervix to hasten delivery. Others, including Willughby, held that labour was safest when left to run its own course, however long that was. He enjoins his readers to wait, because ‘the fruit would fall off it-self, when that it was full ripe’.^51^ What was known to be true about fruit served also for the uterus. His description of the ripening nut inevitably brings us back to Rüff’s ubiquitous birth figures (Figure 2). The peeled membranes enact this ‘natural’ process of birth. The husk, or uterus, slowly peels away, freeing the fetal fruit without need for violence or intervention. Thus these openings, typically understood as an imagined anatomical cut, are perhaps better understood as symbolic of the natural capacity of the uterus to open during labour.

This kind of verdant analogy is visually striking in Rüff’s images, but it is essential to recognise that, for early modern people, it was such a ubiquitous way of thinking, that it would have coloured the way that *all* images of the body were read. William Sherman has described one annotated copy of *The Byrth of Mankynde* held at the Huntington Library. The birth figures in this edition are described by the annotator: ‘These ar ye campes or feeldes of mankynde to be engendred yr in.’^52^ Even images that are, to us, seemingly bare of verdant analogy, spoke to the early modern viewer of the uterus as fertile field, the fetus as both encamped solider and ripe crop. So essential was analogical thinking to early modern body culture that artists did not need to use striking visual analogy for the viewer to interpret an image of the body as a reflection of the natural world.

As well as agricultural, domestic analogy was often used to understand the pregnant body. Fetuses were regularly described in midwifery manuals as miniature people with agency, who lived in a human relation to the uterus, described as a room or cottage. Indeed, it was common in this period to envision the body as a house, in which the uterus was a private chamber or secret box, housing the inaccessible and much prised fetus. Jakob Rüff, for instance, describes how ‘after the third and fourth moneth from the conception, the Infant doth begin to move and stirre himselfe in the wombe, and somewhat to display and stretch out himselfe, and also to enlarge and amplifie his narrow little Cottage’.^53^

This fetus is the same autonomous little being found in so many birth figures, and the uterus his ‘little Cottage’. It is particularly significant that Rüff describes the uterus as a house at the moment of quickening. Quickening, or the first time a mother feels the fetus move, was a significant moment in an early modern pregnancy. Not only was it the best and most trusted indicator of pregnancy, it was also often thought of as the moment of ensoulment, when the fetus went from being a passive thing to an active living being—when it became human. With quickening, a woman would become more confident about her pregnancy, and was likely to be more willing to imagine the fetus as a little person. Rüff describes the fetus suddenly moving, stirring and stretching himself out, like someone waking up. Indeed, as the fetus began to move and kick, the active, independent little child of the birth figure becomes a useful way for the mother to visualise and explain her physical sensations. As the fetus and the belly both grow, too, Rüff describes a process in which the fetus literally stretches out the uterus, creating for himself something more like a little room, and less like a tight covering, and this serves to emphasise the quickened child as an autonomous human with a human-scaled relationship to its living space.

In this period, Mary Fissell shows, the fetus was not just thought of as a miniature human in a domestic environment, he was thought of specifically as a houseguest, and the mother as both housewife and house. Fissell argues that ‘the fetus was imagined as a sort of guest within the mother’s body, and it was her job to provide appropriate hospitality to it, just as she would in her own home’.^54^ This domestic narrative was often used to explain the onset of labour. The uterus, the cottage or room of the fetus, would become insufficient for it as it neared full term. The mother would begin to fail in her ability to give the fetus enough room and enough nourishment, and the amniotic fluid (which was thought at this time to be composed of fetal urine and sweat) would become too oppressive. So, like a disgruntled houseguest dissatisfied with the hospitality provided, the fetus would have to leave. In *The Compleat Midwifes Practice* this process is described: ‘The third [reason for labour] is the narrowness of the place where the infant lies, so that he is forced to seek room other-where, which makes him to break the membranes wherein he was contained, pressing and constraining the mother by the sharpness of those waters, to do her duty for his release.’^55^

This understanding of the mother’s duties of hospitality towards the fetus, and the understanding of the fetus as a human guest with an awareness of his own needs and the ability to leave if they are not met, is reflected in birth figures. The fetuses often look with fixed expressions at the cervix, as if contemplating their escape. Indeed, as often as the maternal body is thought of as a house in this period, it is thought of as a prison. So one Scottish writer, James McMath, describes childbirth as the fetus ‘having thus escaped from its *Prison* through *Nature’s* triple *Gates*, … it appears a new *Guest* upon the World’.^56^ The uterus becomes a prison as the fetus grows, the cervix and vagina the gates it must escape through to become a guest no longer upon the microcosmic maternal body but upon the macrocosmic world.

The personified fetus that we see in birth figures, therefore, was part of a larger system for thinking about the unborn child that helped to explain the internal mysteries of conception, pregnancy and labour. A fetus becoming more person-like, or ensouled, when it quickened helped women to adjust their pictures of their unborn children—from early conceptions that might be false or monstrous, or might simply slip away—to a more viable child. In labour too, the personified fetus helped women to understand their bodily processes. Although it was well understood among midwives that the mother also worked at a labour, a narrative which personified the fetus allowed people to explain difficult deliveries and still births. If the fetus was weak or not ready, a labour would take longer, more so if it had died *in utero*. Thus, the mysteries of lingering labours could be rationalised and even blamed on the strength and willingness of the fetus.

From conception to birth, analogical thinking provided a framework for visualising and understanding the pregnant body. In itself a visually inaccessible and mysterious process, pregnancy could be understood through knowledge of the world. This system allowed people of all skills and conditions to acquire knowledge about and abilities in treating the pregnant and labouring body. Indeed, the birth figure could be seen as an emblem for analogical thinking: an image of the fetus within the uterus, hidden and small inside the body, and at the same time an image of man in the world, the microcosm and the macrocosm all at once. These images indicate that pregnancy was a condition that echoed throughout the spheres of the macrocosm, finding correspondences between body and world that illuminated both. What might be dismissed by a modern mind as simplistic, naïve anatomies are, in their own historical context, visualisations of the lynch-pin of life and creation, the innermost circle in the great concentric spheres that made up the analogical worldview.

## Women Using Birth Figures

This article aims to approach birth figures with what William Sherman calls ‘an awareness of the gap between the author’s words on the page and the meaning particular readers want to derive from them’.^57^ A product of cultural context, each early modern viewer would ‘read’ a birth figure differently, from the trained midwife seeing a ‘practitional key’, to the physician seeing an alchemical flask, to the farmer seeing a field and crop. To conclude this article, I will ask not what authors and artists expected birth figures to communicate, but rather how they were used and understood by those viewers whom birth figures depicted, and whom they were often ostensibly for, but whose experience is now so hard to recover: pregnant women. Almost no direct accounts of how early modern women experienced or understood pregnancy and childbirth exist. Such topics were quite simply not written about, even by those few women who did regularly write about their lives. How birth figures were understood by women is also something almost never recorded. Yet understanding how women understood birth figures is key to establishing how they contributed to and expressed early modern body culture. This final section will use the few extant primary sources as well as wider historical context to try and reconstruct how laywomen used and understood birth figures.

While there are few contemporary records for how birth figures were used, there is evidence to suggest that both midwifery books and their images were regularly brought into the birthing chamber, either by the midwife or by another attendant, perhaps a local literate woman of high status who oversaw the community’s births.^58^ Jennifer Richards, while remaining rightly wary of speculating on a widespread female readership for midwifery manuals because of the lack of material evidence, does present a case for such books being used as sources of information by midwives.^59^ And, given the general popularity of the genre in the period, Elaine Hobby has argued for a general lay readership: ‘It is reasonable to assume that these books were a significant source of information about the body to the wider reading public.’^60^ The success of midwifery manuals, the number of titles and editions published in England in the seventeenth century, suggest that they were generally popular books. This, combined with the fact that many authors explicitly directed their writing to a readership of women and midwives, suggests that they were likely known to and used by women. The rare evidence from Siegemund’s writing supports the supposition that midwifery manuals and their images were seen by women to contain valuable knowledge of childbirth, and could be consulted during a difficult labour.

But if it is hard to track the extent to which women used midwifery manuals, tracking how they used and understood *birth figures* is even more difficult. The midwifery writer Percival Willughby provides the only description I have found not of how birth figures were intended to be used, but of how they were *actually* used by women in the context of the birthing chamber. He describes these images as ‘all the schemes, and various figures, on which midwives look, making their women to think of wonders, by shewing them these pictures of children, assuring them, that, by these, they bee directed, and perfected, and much enlightened in the way of midwifery.’^61^ Willughby describes midwives bringing midwifery manuals into the birthing chamber and showing the figures to the labouring women and other attendants. These images function, he says, as a kind of visual certification for the midwife’s skills—they show the laywomen the special knowledge of the body and the fetus that the midwife has. While for some midwives, birth figures may have functioned as ‘practitional’ keys, for many women, it seems, they were images with a different message: symbols of expertise and rarefied knowledge, offering comfort in a time of pain and uncertainty.

At the beginning of this article I discussed briefly the understanding of birth figures as images of ‘maternal erasure’. Karen Newman, for instance, has argued that these images ‘suppress completely fetal dependence on the female body by graphically rendering that body as a passive receptacle, the scriptural woman as “vessel”’.^62^ But this argument is based on her understanding of early modern birth figures as precursors to modern fetal imagery and its uses and interpretation in abortion rights debates. For some male and medically educated early modern viewers, birth figures *may* have served to deny the importance of the maternal body, to set up a direct link between male physician and male fetal patient, but this was far from the case for most viewers. In the early modern period, the labouring woman was still understood to be the central agent in birth: and, as Laura Gowing has argued, midwives expected to assist ‘active, hard-working mothers’.^63^ Moreover, the labouring woman was understood as an authority on her body, and midwives worked *with* their clients, listening to their descriptions of their bodily sensations and often deferring to them on issues of medical treatment and birthing position. It was not until the nineteenth century that the labouring woman became a ‘patient’ in the modern sense, subject to the directions of the male practitioner, regularly confined to a reclining position on a bed and, after the mid-nineteenth century, perhaps etherised. For early modern women, the female body could not be wholly denied or disempowered; it was the central agent in birth. The ‘excision’ of the maternal body from birth figures, therefore, was not an excision at all. Rather, as Baxandall has argued, ‘The best paintings often express their culture not just directly but complementarily, because it is by complementing it that they are best designed to serve public needs: the public does not need what it has already got.’^64^ Women already had the female body and knew its importance in generation and birth. What birth figures gave them was a window onto the *interior* of that present, primary, but opaque and mysterious body. It gave them what they did *not* have: a view inside.

Lianne McTavish has noted that birth figures seem to communicate ‘discrepant messages, alluding to both danger and health, dismemberment and vitality’.^65^ These are images of dangerous malpresentations; they illustrate cases in which pain and the risk of death are greatly increased, in which fetuses lie in positions in which they *cannot* be born, and in which they may well die undelivered. Yet the often-serene facial expressions, the floral openings of Rüff’s images, and the way the fetuses seem to slip, fall, swim or be poured from the uterus evoke natural, successful and non-interventionist labour. This inherent ambiguity is perhaps a key to why birth figures were so popular. For the midwife, they gave valuable information about fetal presentation in a crisis situation. For the learned viewer, they engaged in wider philosophical frameworks for understanding creation and the world. But for labouring women and their attendants, these images were perhaps most commonly symbols of expert knowledge, a picture of the fetus inside, and, importantly, an expression of fetal health and liveliness.

As well as reassuring the labouring woman of the special skills of her midwife, birth figures present a positive and encouraging view of the *child*. On the most fundamental level, they present a picture of what could not be seen and what was, until birth, always uncertain. This was a period in which even having a ‘great belly’ did not guarantee a child. Tumours and swellings sometimes fooled women and their midwives; and even if the woman *was* pregnant, she could have engendered a *mola* or, even worse, a monster. Only with birth did the child become a certainty. In this context, a birth figure may well have been a deeply reassuring image of exactly what the labouring woman was hoping for: a healthy, active, well-grown boy child.

Understanding the birth figure as a reassuring epitome of fetal health and well-being allows us to understand some of the representational conventions employed. These images do not show us fragile, scrawny neonates, but rather chubby, active toddlers—these fetuses are represented as *putti.* McTavish has suggested that fetuses were represented as *putti* because that is what artists were trained to draw.^66^ While this is certainly likely, I suggest that the use of *putti* was not simply an accident of style, but an active choice that made birth figures attractive to early modern viewers. First, all the fetuses shown in these images are boys. Boys in the seventeenth century were not only typically socially and culturally preferred, they were also considered biologically more perfect. As Thomas Laqueur argues with the ‘one-sex model’, until the mid-eighteenth century, ‘there had been one basic structure for the human body, and that structure was male’. ^67^While has become clear, since the publication Laqueur's *Making Sex* in 1990, that the one-sex model did not universally apply to early modern understandings of the body and gender, it is an idea expressed in many midwifery manuals of the period. The midwifery author Jane Sharp, for instance, explains how ‘The whole Matrix considered with the stones and Seed vessels, is like to a mans yard and privities, but Mens parts for Generation are compleat and appear outwardly by reason of heat, but womens are not so compleat, and are made within by reason of their small heat.’^68^

The female body was, within this framework, nothing less than an inferior and imperfect version of the male: a body lacking the heat to extrude its genitalia. Because the female body was inferior rather than dimorphic, the conception of a boy proved that the child was strong and healthy. Moreover, it also stood as testament to the vigour and health of both parents, who were able to produce a boy child. Many midwives took this preference for boys even further, arguing, as Jane Sharp did, that ‘a Boy is sooner and easier brought forth than a Girle; the reasons are many, but they serve also for the whole time she goes with Child, for women are lustier that are with Child with Boys, and therefore they wull be better able to run through with it’.^69^ Thus an image of a boy child was, for the viewing mother, a picture of her own health and that of her child, as well as an easier labour.

Furthermore, the representation of fetus as *putto* was in itself a message of reassurance to the maternal viewer. John Heilbron has identified that *putti* were regularly employed in early modern scientific images to ‘domesticate’ or make approachable new scientific experiments and techniques. He argues that ‘Playful small angels demonstrating the laws of optics or working an air pump might indicate the harmlessness, innocence, and correctness of experimental natural philosophy.’^70^*Putti* not only associated science with pleasure, amusement, youthful mischief and love, they also embodied the ideal infallible experimenter. The same qualities serve to present an idealised view of childbirth to the lay viewer in which the fragile, intensely vulnerable fetus is represented as an embodiment of health, activity, wisdom, playfulness, even angelic immortality. So, what to a midwife was a catalogue of complications, could be read by laywoman as a series of youthful hijinks, a positive and encouraging view of a healthy boy child, with plenty of room in the uterus and plenty of strength and agility with which to get out.

Birth figures made the early modern viewer, in Willughby’s words, ‘think of wonders’, giving a miraculous view of the opaque belly, a peek at the inaccessible and (usually) much desired child and a visualisation of the mysterious and miraculous processes of conception and fetal growth. Yet these were not just images of wonder, but images that actively produced wonders. In the early modern period, it was widely understood that strong impressions made on the mother during pregnancy would imprint on and form the child. As Jane Sharp wrote in her manual, ‘sometimes the mother is frighted or conceives wonders, or longs strangely for things not to be had, and the child is markt according to it’.^71^ This could lead a mother to accidentally mark or disfigure her child, or even to engender a monster, but maternal imagination could also be employed for positive ends.^72^ In Italy, for example, *deschi da parto*, painted birth-trays given as gifts to pregnant women, would sometimes depict healthy, handsome boy children. Katharine Park has argued that ‘by gazing attentively at these objects, mothers could literally shape their offspring, raising their chances of producing a well-formed son’.^73^ These *deschi* bear some striking resemblances to Rösslin’s and Rüff’s birth figures. Both tray and birth figure tend to depict an idealised child; emphasising health, strength, beauty and, of course, maleness. Read analogically, *deschi* could even depict the fetus in the body, the rim of the tray delimiting the uterus, the verdant landscape in which the child often stands, an expression of the maternal bodily environment. But the *descho* is not simply an image of encouragement, it is a complete expression of health in pregnancy that could be gazed at and *replicated within* the body. Birth figures, cheap and widely accessible across Europe, arguably served the same function. Inside and without the birthing chamber, these little images were ones of positive power. For the early modern viewer, such images did not simply represent the body: the links between body and image were closer and more complicated. The image is a rendering of the body, but through a process of looking and imagining, the body also becomes a rendering of the image. And so birth figures enter the complex web of early modern body culture, both expressing and forming that culture; both reflecting and creating the ways in which people envisioned the pregnant interior.

Birth figures are images that, in their multiple interactions with body culture, challenge our assumptions about the body as a subject for historical study. They pull together types of knowledge about the body that are more typically treated separately and they bring to the fore types of popular body knowledge that are often eclipsed by the study of medical innovations. To look at these images is to be confronted with what seem to us to be contradictions—images of medical practice, influenced by anatomy, that are also verdant and analogical, alchemical and humoral, even wondrous. Only by looking at these images as working simultaneously in multiple registers can we reconcile these seeming contradictions and gain a more thorough understanding of early modern body culture.

This multiplicity, combined with their remarkably wide viewership, which makes it so hard to ascribe just *one* function or reading to these images, makes them valuable sources for looking at a culture that was essentially inclusive, imaginative and multifarious in its thinking about the body. Birth figures remind us that this was a period in which learned and vernacular, old and new, male and female ways of knowing met, interacted and mingled at the site of the pregnant body. Just as the early modern woman could look at a birth figure as a window onto her own mysterious bodily interior, so we can approach these images as windows onto the rich and complex body culture of early modern England.

## Footnotes

1 Lianne McTavish, *Childbirth and the Display of Authority in Early Modern France* (Aldershot: Ashgate, 2005), 191; Lyle Massey, ‘Pregnancy and Pathology: Picturing Childbirth in Eighteenth-Century Obstetric Atlases’, *The Art Bulletin*, 2005, 87, 73–91, 76; Harold Speert, MD, *Obstetrics and Gynecology: A History and Iconography* (San Francisco: Norman Publishing, 1994), 153; K. B. Roberts and J. D. W. Tomlinson, *The Fabric of the Body: European Traditions of Anatomical Illustration* (Oxford: Clarendon Press, 1992), 22.

2 Eucharius Rösslin, *The Byrth of Mankynde, Otherwyse Named the Womans Booke*, T. Raynalde (trans.), (London: Thomas Raynalde, 1545); Elaine Hobby, ed., *The Birth of Mankind* (Farnham: Ashgate, 2009), xxx.

3 While this article will focus on how such images were received and understood in England, it is important to note that the *same* birth figures were reproduced in books all over Europe, in Latin and in various vernaculars. Thus, while each country had a different culture of midwifery, they were also engaging in a new kind of visual culture that crossed national borders. As such, the arguments made in this article will be of relevance to studies of midwifery in France, Germany, the Netherlands, Switzerland and even further afield.

4 The artist and engraver Martin Caldenbach, a pupil of Albrecht Dürer, is known to have produced the birth figures for Rösslin’s original German manual, see Peter M. Dunn, ‘Eucharius Rösslin of Germany and the Rebirth of Midwifery’, *Archives of Disease in Childhood. Fetal and Neonatal Edition*, 1998, 79, 77–8. Jennifer Spinks attributes the original woodcuts from Jakob Rüff’s *Trostbüchle* to Jos Murer, see Jennifer Spinks, ‘Jakob Rueff’s 1554 *Trostbüchle*; a Zurich Physician Explains and Interprets Monstrous Births’, *Intellectual History Review*, 2008, 18, 41–59. However, both sets of images were widely copied and reproduced by a host of anonymous draughtsmen, printmakers and publishers. For ease of reference, and in recognition of the collaborative nature of such images, in this article I will refer to birth figures by the name of the author of the book in which they were originally printed.

5 See Jacques Guillemeau, *Child-Birth, or the Happy Deliverie of Women* (London: A. Hatfield, 1612); Ambroise Paré, *The Workes of that Famous Chirurgion Ambrose Parey,* Th. Johnson (trans.) (London: Th. Cotes and R. Young, 1634); [Thomas Chamberlayne et al.], *The Compleat Midwifes Practice: In the Most Weighty and High Concernments of the Birth of Man* (London: Nathaniel Brooke, 1656); Jane Sharp, *The Midwives Book: or the Whole Art of Midwifry Discovered* (London: Simon Miller, 1671); James Wolveridge, *Speculum Matricis: or, the Expert Midwives Handmaid* (London, 1671); and [James Wolveridge], *The English Midwife Enlarged* (London: Rowland Reynold, 1682).

6 The only other kind of birth figure that I have found published in England prior to the publication of François Mauriceau’s manual in 1672, is a set found in both James Cooke, *Mellificium Chirurgiae* (London: Samuel Cartwright, 1648) and William Sermon, *The Ladies Companion, or the English Midwife* (London: Edward Thomas, 1671).

7 Roberts and Tomlinson, *The Fabric of the Body*, used the term ‘bottled up’ on p. 22 and ‘bizarre portrayals’ on p. 122. For ‘wildly fantastic pictures’, see Ludwig Choulant, *History and Bibliography of Anatomic Illustration*, Mortimer Frank (trans. and ed.) (Cambridge, MA: Maurizio Martino, 1993), 74.

8 See, for example, Speert, *Obstetrics and Gynecology.*

9 Eve Keller, *Generating Bodies and Gendered Selves: The Rhetoric of Reproduction in Early Modern England* (Seattle: University of Washington Press, 2007), 136; Laura Gowing, *Common Bodies: Women, Touch and Power in Seventeenth-Century England* (New Haven, CT and London: Yale University Press, 2003), 122–7; and Karen Newman, *Fetal Positions: Individualism, Science, Visuality* (Stanford, CA: Stanford University Press, 1996), 33.

10 Mary Fissell, *Vernacular Bodies: The Politics of Reproduction in Early Modern England* (Oxford: Oxford University Press, 2004), 150 and Hobby, *The Birth of Mankind*, xvii.

11 McTavish, *Childbirth and the Display of Authority*, 179.

12 Michael Baxandall, *Painting and Experience in Fifteenth-Century Italy* (Oxford: Oxford University Press, 1988), preface.

13
*Ibid*., 152.

14 Barbara Duden, *The Woman Beneath the Skin: A Doctor’s Patients in Eighteenth-Century Germany* (Cambridge, MA: Harvard University Press, 1991), 5. In this article I use the term ‘body culture’ to refer to the idea, proposed by Duden, of a body that is constructed by a society’s culture, rather than being a ‘natural’ and atemporal constant.

15
*Ibid*., 31.

16 Fissell, ‘Hairy Women and Naked Truths: Gender and the Politics of Knowledge in “Aristotle’s Masterpiece”’, *The William and Mary Quarterly*, 2003, 60, 43–74, 74.

17 Sachiko Kusukawa, *Picturing the Book of Nature: Image, Text, and Argument in Sixteenth-Century Human Anatomy and Medical Botany* (Chicago and London: The University of Chicago Press, 2012), 2.

18 Gianna Pomata, 2011. ‘Observation Rising: Birth of an Epistemic Genre, 1500–1650’, in Loraine Daston and Elizabeth Lunbeck, eds, *Histories of Scientific Observation* (Chicago and London: The University of Chicago Press, 2011), 45–80, at 48, 53.

19 Monica Green, ‘The Sources of Eucharius Rösslin’s “Rosegarden for pregnant women and midwives” (1513)’, *Medical History*, 2009, 53, 167–92 and McTavish, *Childbirth and the Display of Authority*, 33.

20 For the 1545, 1552 and 1560 editions, the Vesalian anatomies were actually cut from sheets produced for Thomas Geminus’s anatomical text *Compendiosa Totius Anatomie Delineatio* (1545) and pasted into *The Byrth of Mankynde*. Thereafter, specific plates were produced for the book. For a full account see LeRoy Crummer, ‘The Copper Plates in Raynalde and Geminus’, *Proceedings of the Royal Society of Medicine,* 1926, 20, 53–56.

21 Kusukawa, *Picturing the Book of Nature*, 195–9.

22 Rösslin, *The Byrth of Mankynde*, B3 r.

23 Duden, *The Woman Beneath the Skin*, 106.

24 Michel Foucault, *The Birth of the Clinic: An Archaeology of Medical Perception*, A. M. Sheridan (trans.) (London and New York: Routledge, 2003).

25 Lorraine Daston and Peter Galison, *Objectivity* (New York: Zone Books, 2010), 113.

26 Rösslin, *The Byrth of Mankynde*, B3 v.

27 Sarah Stone, *A Complete Practice of Midwifery* (London: T. Cooper, 1737), xv.

28 ‘Practitional’ is a term I use here to indicate images that represent the knowledge of practising midwives just as ‘anatomical’ images represent the knowledge of anatomists.

29 See Percvial Willughby, *Observations in Midwifery* (Wakefield: S.R. Publishers, 1972), 276; Stone, *Complete Practice*.

30 Alice Thornton, *The Autobiography of Mrs. Alice Thornton, of East Newton, Co. York.* (Durham: Surtees Society, 1875), 96.

31 Adrian Wilson, ‘A Memorial of Eleanor Willughby, A Seventeenth-Century Midwife’, in Lynette Hunter and Sarah Hutton, eds, *Women, Science and Medicine 1500–1700* (Trupp: Sutton Publishing, 1997), 138–77, 143–8.

32 ‘Reduction to the head’ is advised by both Rösslin and Rüff.

33 It is possible that some midwives were using podalic version before this time, but it is generally accepted that Paré was the first to publish the technique. See Janet Doe, *A Bibliography, 1545–1940, of the Works of Ambroise Paré, 1510–1590* (Amsterdam: Gérard Th. van Heusden, 1976), xiii–xiv.

34 Hannah Newton, ‘“Nature Concocts and Expels”: The Agents and Processes of Recovery from Disease in Early Modern England’, *Social History of Medicine*, 2015, 28, 465–86.

35 Jakob Rüff, *The Expert Midwife or An Excellent and Most Necessary Treatise on the Generation and Birth of Man* (London: E.G., 1637), 81.

36 Jennifer Richards, ‘Reading and Hearing The Womans Booke in Early Modern England’, *Bulletin of the History of Medicine*, 2015, 89, 434–62, 461.

37 Siegemund’s testimony is discussed in this article because it is the only instance I have found of a midwife of the seventeenth century describing how she used birth figures. It is such rare evidence that I believe it is worth examining, despite fact that Siegemund worked and published in Germany and not England. The birth figures available would have been much the same in both countries.

38 Justine Siegemund, *The Court Midwife*, L. Tatlock (trans.) (Chicago and London: The University of Chicago Press, 2005), 90.

39
*Ibid*., 130.

40 Michel Foucault, *The Order of Things: An Archaeology of the Human Sciences* (London and New York: Routledge, 2005), 19.

41
*Ibid*.

42 Thomas Laqueur, *Making Sex: Body and Gender from the Greeks to Freud* (Cambridge, MA and London: Harvard University Press, 1990), 131.

43 Laurinda Dixon, *Perilous Chastity: Women and Illness in Pre-Enlightenment Art and Medicine* (Ithaca and London: Cornell University Press, 1995), 106.

44 Paré, *The Workes*, 885.

45 William Newman, *Promethean Ambitions: Alchemy and the Quest to Perfect Nature* (Chicago and London: University of Chicago Press, 2004).

46
*Ibid*., 164–237.

47 Michael Stolberg, *Uroscopy in Early Modern Europe* (Farnham: Ashgate, 2015), 119.

48
*The Casebooks Project*, http://www.magicandmedicine.hps.cam.ac.uk/.

49 Willughby, *Observations in Midwifery*, 276.

50
*Ibid*., 276–7.

51
*Ibid*., 7.

52 William Sherman, *Used Books: Marking Readings in Renaissance England* (Philadelphia: University of Philadelphia Press, 2008), 61.

53 Rüff, *The Expert Midwife*, 62.

54 Fissell, *Vernacular Bodies*, 32.

55 [Chamberlayne *et al*.], *The Compleat Midwifes Practice*, 73.

56 James McMath, *The Expert Mid-wife: A Treatise of the Diseases of Women with Child, and in Child-bed* (Edinburgh: George Mosman, 1694), 119–20.

57 Sherman, *Used Books*, 22.

58 Jennifer Wynne Hellwarth, ‘“I Wyl Wright of Women Prevy Sekenes”: Imagining Female Literacy and Textual Communities in Medieval and Early Modern Midwifery Manuals’, *Critical Survey*, 2002, 4, 44–63.

59 Richards, ‘Reading and Hearing’.

60 Elaine Hobby, ‘“Secrets of the female sex”: Jane Sharp, the Reproductive Female Body, and Early Modern Midwifery Manuals’, *Women’s Writing*, 2007, 8, 201–12, p. 201.

61 Willughby, *Observations in Midwifery*, 341.

62 Newman, *Fetal Positions*, 33.

63 Gowing, *Common Bodies*, 168.

64 Baxandall, *Painting and Experience*, 48.

65 McTavish, *Childbirth and the Display of Authority*, 193.

66
*Ibid.*, 192.

67 Laqueur, *Making Sex*, 10.

68 Sharp, *The Midwives Book*, 37.

69
*Ibid*., 168.

70 John Heilbron, ‘Domesticating Science in the Eighteenth Century’, in W. R. Shea, ed., *Science and the Visual Image in the Enlightenment* (Canton, MA: Science History Publications, 2000), 1–24, 1.

71 Sharp, *The Midwives Book*, 117–18.

72 Frances Gage, *Painting as Medicine in Early Modern Rome: Giulio Mancini and the Efficacy of Art* (Pennsylvania: The Pennsylvania State University Press, 2016), 87–199.

73 Katharine Park, *Secrets of Women: Gender, Generation, and the Origins of Human Dissection* (New York: Zone Books, 2010), 145.

## Notes

Rebecca Whiteley is a PhD Candidate in the History of Art department at University College London, where she is working on early modern print images of the pregnant body and unborn child. Her research interests include early modern print and visual cultures, social history and histories of science and medicine. She completed a research fellowship at the Huntington Library in the summer of 2016.

